# GnRH antagonist weakens endometrial stromal cells growth ability by decreasing c-kit receptor expression

**DOI:** 10.1186/s12958-021-00886-y

**Published:** 2022-02-04

**Authors:** Ding-Fei Xu, Pei-Pei Liu, Lu Fan, Qi Xie, Zhi-Qin Zhang, Li-Qun Wang, Qiong-Fang Wu, Jun Tan

**Affiliations:** 1 Jiangxi Medical College, Nanchang University, Nanchang, Jiangxi 330006 P. R. China; 2 Reproductive Medicine Center, Maternal and Child Health Hospital Affiliated to Nanchang University, Jiangxi Maternal and Child Health Hospital, Nanchang, Jiangxi 330006 P. R. China; 3 Department of Reproductive Health, Maternal and Child Health Hospital of Nanchang University, Jiangxi maternal and child health hospital, Nanchang, Jiangxi 330006 P. R. China

**Keywords:** GnRH-ant, C-kit receptor, ESCs, Growth ability

## Abstract

**Background:**

Several surveys have reported that patients treated with gonadotropin-releasing hormone antagonist (GnRH-ant) protocol showed a significantly lower rate of implantation and clinical pregnancy compared to GnRH agonist (GnRH-a) protocol during in vitro fertilization-fresh embryo transfer. Subsequent studies imputed this poor outcome to the negative effects of GnRH-ant on endometrial receptive. However, the mechanisms were not fully understood.

**Methods:**

The clinical data of 2815 patients undergoing fresh embryo transfer in our center were analyzed. Human endometrial stromal cells (ESCs) from healthy women undergoing elective pregnancy termination of a normal pregnancy at 8–10 weeks gestation were treated with GnRH-analogs or imatinib (c-kit receptor inhibitor). CCK8 and Flow cytometry were used to investigated the growth ability of ESCs. Immunofluorescence staining and western blot was used to detected the target proteins.

**Results:**

The clinical data showed that the endometrial thickness on HCG Day were significantly lower in GnRH-ant group. Although no difference of embryo quality in these two groups, GnRH-ant group showed remarkably decreased rate of HCG positive, embryo implantation and pregnancy. Moreover, GnRH-ant significantly reduced the proliferation and induced the apoptosis of ESCs. Furthermore, the expression and activation of c-kit receptor, which played pivotal roles during embryo implantation, were observably decreased by GnRH-ant. Inhibiting the activation of c-kit by imatinib remarkably suppressed the proliferation and promoted the apoptosis of ESCs. Additionally, the phosphorylation of AKT and expression of Cyclin D1, which were closely related with cellular growth, were distinctly lessened after treating with imatinib.

**Conclusions:**

In summary, our study showed that GnRH-ant weakened the activization of c-kit receptor by decreasing its expression, causing the impaired growth ability of ESCs. Our findings provided a new insight into the effects of GnRH-ant on endometrium.

**Supplementary Information:**

The online version contains supplementary material available at 10.1186/s12958-021-00886-y.

## Background

Gonadotropin-releasing hormone antagonist (GnRH-ant), which could competitively bind with GnRH receptor (GnRHR) and reversibly inhibited hypothalamic-pituitary-gonadal axis, has been used in assisted reproduction for several decades from the first generation to the recent third generation [1]. Based on the superiority of convenience, flexibility and safety, GnRH-ant protocol has been one of the most commonly therapeutic schedules in in vitro fertilization-embryo transfer (IVF-ET) [2]. However, the unsatisfactory clinical outcomes of this protocol, compared to GnRH-agonist (GnRH-a) protocol, has restrained its promotion and application in clinic. Many clinical investigations have reported lower fresh embryo implantation and pregnancy rates in GnRH-ant protocol than those in GnRH-a protocol [3–5]. Similarly, Bukulmez’s research also showed a lower HCG positive and clinical pregnancy rate in GnRH-ant protocol when compared to GnRH-a protocol within no difference of embryo quality between these two protocols [6]. Additionally, decreased clinical outcomes from GnRH-ant protocol were only obvsered in fresh embryo transfer but not in freezing embryo transfer [7], suggesting that the adverse effects of GnRH-ant on endometrium might be the key reason.

In fact, many researches have demonstrated that the endometrial thickness could be an important factor which influenced the pregnancy after embryo transfer [8–11]. Patients with endometrial thickness less than 8 mm experienced lower implantation and pregnancy rate [12]. Significantly, recent studies have proved that patients treated with GnRH-ant protocol developed a remarkably thinner endometrial thickness than patients treated with GnRH-a protocol [13, 14]. These results implied that one of the adverse effects of GnRH-ant on endometrium was to weaken endometrial thickness. Nonetheless, the related molecular mechanism was still unclear.

Previous studies have found the expression of c-kit receptor on embryo and endometrium [15], suggesting the crucial role of c-kit receptor in the process of animal embryo implantation. C-kit receptor belongs to RTK type III family which could be stimulated by stem cell factor (SCF). Activated c-kit receptor was associated with multiple kinds of biological events, including proliferation, differentiation, migration and apoptosis, in many types of cells [16, 17]. Our previous study reported the expression of c-kit receptor on human embryo and demonstrated that added exogenous SCF to activate c-kit receptor significantly promoted embryo development [18]. Recently, we found the expression of c-kit receptor on human endometrial stromal cells (ESCs). Based on the regulatory function on the growth of many kinds of cells, we speculated that c-kit receptor might also influence the growth of human ESCs. Furthermore, whether its regulatory effect is associated with GnRH-ant is still unknown.

Therefore, the present study was conducted to explore the effects of GnRH-ant and c-kit receptor on the growth of human ESCs, and to investigated the regulatory relationship between GnRH-ant and c-kit receptor.

## Methods

### Reagents

The following reagents were used in this study: rabbit anti-GnRHR (Abcam, UK), mouse anti-c-kit (CST, USA), rabbit anti-c-kit (CST, USA), rabbit anti-phospho-c-kit (CST, USA), rabbit anti-AKT (CST, USA), rabbit anti-phospho-AKT (CST, USA), rabbit anti-Cyclin D1 (CST, USA), mouse anti-β-actin (Santa Cruz, USA), Donkey anti rabbit Alexa flour 488 (Thermo Fisher Scientific, USA), Donkey anti rabbit Alexa flour 594 (Thermo Fisher Scientific, USA), GnRH-a (Decapeptyl; Ferring), GnRH-ant (Cetrotide; Serono) and Imatinib (Biovision, USA).

### Patients

The present study retrospectively analyzed the clinical data of 2815 patients undergoing fresh embryo transfer in Reproductive Medicine Center of Jiangxi Maternal and Child Health Hospital from Jan 2016 to Dec 2020. The reasons of infertility were oviduct diseases and/or male factors. The inclusion criteria for all patients included age ≤ 37 years, body mass index (BMI) of 15–25 kg/m^2^, 1.1 < anti-Müllerian hormone (AMH) < 5.0, 5 ≤ antral follicle count (AFC) ≤ 20, and less than twice IVF-ET experiences. Women with a history of the following procedures or disorders were excluded: uterine malformation, ovarian surgery, radiotherapy or chemotherapy, premature ovarian failure, ovarian dysfunction, adenomyosis, polycystic ovarian syndrome, thyroid dysfunction, recurrent implantation failure (failed to achieve a pregnancy more than three times), submucosal fibroids, intrauterine adhesion, hydrosalpinx, and patients (women or men) with abnormal chromosomes.

### Study design and groups

Two thousand eight hundred fifteen patients were classified as GnRH-ant group (563) and GnRH-a group (2252) according to the different protocols. In GnRH-ant group, recombinant human FSH (rhFSH, Merck-Serono, German) treatment began on day 2 or 3 of the menstrual cycle. The initial dosage (112.5–225 IU/day) was determined based on age, BMI, AFC, and AMH. The daily dose of rhFSH was adjusted according to ovarian response as monitored by ultrasonography and serum estradiol (E_2_) levels. GnRH antagonist (Cetrorelix, Merck Serono, Switzerland) at a daily dose of 0.25 mg was started when the largest follicle exceeded 12 mm. Both GnRH antagonist and rhFSH were stopped and a single injection of 6000–8000 IU of hCG (Merck-Serono, German) was administered when the dominant follicle was ≥19 mm in diameter or at least 2 follicles were ≥ 18 mm in diameter. Oocyte retrieval was performed 36–40 h later under transvaginal ultrasound guidance. In GnRH-a group, a standard full dose of gonadotropin-releasing hormone agonist (administered as a one-time dose of 3.75 mg, GnRH agonist, Ipsen, France) was used on the second day of menstrual cycle for down regulation. Pituitary down regulation (Endometrial thickness ≤ 5 mm, serum FSH < 5 mIU/mL, LH < 5 mIU/mL, E_2_ < 50 pg/mL) was confirmed with transvaginal ultrasound and endocrine examination after 28–30 days. The initial dosage (112.5–225 IU/day) was determined based on age, BMI, AFC, and AMH. The dose of rhFSH were adjusted according to ovarian response as monitored by ultrasonography and serum estradiol levels. The HCG trigger process and oocyte retrieval was the same as described above.

HCG positive rate: HCG concentration was defined by measuring blood 14 days (embryo at day 3) or 12 days (blastocyst) after embryo transfer, and the threshold concentration of HCG positive was > 5 mIU/mL. The HCG positive rate refers to the number of HCG positive patients divided by the total number of patients. Clinical pregnancy rate: fetal heartbeat was used to define clinical pregnancy by ultrasonography 1 month after embryo transfer. The clinical pregnancy rate refers to the number of clinical pregnancy patients divided by the total number of patients. Implantation rate: the number of embryos with fetal heartbeat divided the total number of all transferred embryos.

### Embryo assessment

The embryo assessment criteria were executed as the previously described [18]. A good quality embryo should consist of 7–9 blastomeres with a uniform size, and the fragment proportion should be less than 20% at day 3 for human 2PN embryos after fertilization. The good quality embryo rate refers to the number of good quality embryos divided by the total number of all embryos. Blastocyst formation was determined and graded by using the system of Gardner and Schoolcraft [19]. The blastulation rate refers to the number of blastocysts divided by the total number of all embryos. The good quality blastulation rate refers to the number of good quality blastocyst divided by the total number of all blastocysts. The assessment was made in a blinded manner by two embryologists. Embryo transfer was conducted on day 3 for embryos or day 5 and day 6 for blastocysts after oocyte retrieval.

### Primary human ESCs isolation, culture and treatment

Primary human decidual ESCs were isolated from the decidual tissue of healthy multipara women (aged 25–32 years) undergoing elective surgical termination of a normal pregnancy at 8 to 10 weeks of gestation. The informed consents from all patients were obtained before the initiation of this study. According to the standard protocol [20, 21], the human decidual tissue was minced and treated with type IV collagenase and DNase type I in a shaking water bath at 37 °C for 90 min. The cell digest was then passed through a 70 μm filter, both decidual stromal and epithelial cells were collected. Then, decidual stromal cells were separated from epithelial cells with a 45 μm filter. The stromal cells were subsequently pelleted by centrifugation at 1000 rpm for 5 min. The cell pellets were washed once, resuspended, and cultured in Dulbecco modified Eagle medium containing 25 mM glucose, Lglutamine, antibiotics and supplemented with 10% fetal bovine serum at 37 °C. ESCs was confirmed by detecting the expression of vimentin protein via immunohistochemical analysis (Supplementary Fig. 1). The cells were used after reaching 70–80% confluence. ESCs were treated for 120 h with different concentrations of GnRH-a, GnRH-ant (10^− 8^, 10^− 5^, and 2 × 10^− 5^ mol/L) or imatinib (0 μM, 4 μM, 8 μM, 16 μM, 24 μM, 32 μM). Cells were subsequently collected for cell proliferation assay, flow cytometry and western blot.

### Cell proliferation assay

Five thousand cells/well were planted into 96-well plates in quintuplicate wells. Normally, cells were placed in the culture medium with different concentrations of GnRH analogs for 24, 48, 72, 96 and 120 h, a total of 10 μL CCK8 reagent (APExBio, USA) was added to each well. After incubating for 2 h at 37 °C, the absorbance at 450 nm per well was measured using microplate reader. Each group of experiments was repeated three times.

### Flow cytometry

The suspended cells were collected by centrifugation. Centrifuge 1000 g, centrifugation time 5 min at 2–8 °C. Cultured cells needed to be digested with EDTA-free trypsin, then terminated with serum-containing medium, centrifuged at 1000 g for 5 min, supernatant removed, and washed with PBS resuspension. After centrifugal precipitation, the PBS was resuspended, transferred into the flow tube, washed once with PBS, centrifuged at 1000 g for 5 min, and the supernatant was discarded. Cells were suspended with 400 UL × Annexin binding solution at a concentration of approximately 1 × 10^6^ cells/ml. Five UL Annexin V-FITC staining solution (BestBio, China) was added to the cell suspension, gently mixed and incubated for 15 min at 2–8 °C in the dark. After adding 10 UL PI staining solution, gently mixed and incubated for 15 min at 2–8 °C in the dark. Immediately detected by flow cytometry. Each group of experiments was repeated three times.

### Immunofluorescence staining

The methods were described previously [22]. Cells were fixed with 4% paraformaldehyde at 4 °C for 10 min and washed with PBS three times. The cells were sealed with 2% triton-100 solution at room temperature for 30 min. The primary antibody diluted by blocking solution was added and incubated overnight at 4 °C. The next day, cells were washed with PBS three times, 5 min each time. The fluorescent secondary antibodies were added and incubated for 1 h at room temperature in the dark, then the cells were washed with PBS for 3 times, 5 min each time. Fluoroshield mounting medium (containing DAPI) with 1:10 dilution was added and stored at 4 °C in the dark for fluorescence microscopy observation.

### Western blot

Total proteins were extracted from cells using the RIPA lysis buffer containing protease inhibitors (Applygen, China) and phosphatase inhibitors (Sigma, USA). The protein concentrations were determined by NanoDrop 2000c spectrophotometer using BCA protein assay kit (Applygen, China). After loading equal amount of protein samples, SDS-PAGE (12% sodium dodecyl sulfate polyacrylamide gel electrophoresis) was performed. The proteins were then transferred to a PVDF membrane (Merck-Millipore, USA). After blocking with Tris buffered saline containing 0.05% Tween-20 (TBST) and 5% non-fat dry milk or 5% BSA for 1 h, the membrane was incubated with corresponding antibodies at 4 °C overnight, washed in TBST, followed by incubation with the corresponding horseradish peroxidase-conjugated secondary antibodies for 1 h. Visualization of the proteins was detected with ECL chemiluminescence. Beta-actin was used as a loading control. The intensity values were assessed and analyzed with Image J software. (*n* = 4 for per lane).

### Statistical analysis

Statistical analyses were conducted by using SPSS 24.0 software (SASInstitute Inc.), and all data were expressed as means ± standard errors of the means (s.e.m.s) or percentage (%). Results among experimental groups were analyzed by student’s t-test or one-way ANOVA. For all tests, *P*-value < 0.05 was considered statistically significant.

## Results

### General characteristics

No differences were found in general characteristics between these two groups (Table 1).

**Table 1:** Comparison of the general information of the two groups of patients

	GnRH-ant (*n* = 563)	GnRH-a (*n* = 2252)	*P*-value
Age (y)	30.99 ± 4.55	31.01 ± 4.2	0.9463
BMI (Kg/m^2^)	21.7 ± 5.35	21.67 ± 3.96	0.9054
Duration of infertility (y)	4.22 ± 3.26	4.24 ± 3.1	0.9202
Antral follicle count (n)	10.94 ± 3.96	10.95 ± 3.71	0.9559
Endometrial thickness (mm)	7.03 ± 2.3	7.08 ± 2.19	0.9508
AMH (ng/mL)	2.76 ± 1.04	2.77 ± 1.04	0.9292
Basal FSH (mIU/mL)	6.19 ± 2.28	6.48 ± 1.01	0.7619
Basal E_2_ (pg/mL)	66.86 ± 16.07	57.28 ± 23.25	0.2600
Basal P (ng/mL)	1.2 ± 3.33	1.55 ± 4.26	0.7954
Basal LH (mIU/mL)	4.44 ± 3.79	4.49 ± 3.45	0.7901

Note: The data were expressed as mean ± SD

Abbreviations: *BMI* Body Mass Index, *AMH* anti-mullerian hormone, *FSH* follicle-stimulating hormone, *LH* luteinizing hormone, *E*_2_ estradiol, *P* progesterone

### Comparison of the IVF outcomes between GnRH-ant and GnRH-a groups

Firstly, we compared the effects of ovulation promotion between GnRH-ant and GnRH-a groups (Table 2). Among the two groups, the levels of E_2_ and P on HCG day were similar (*P > 0.05*), but the initial dose of gonadotropin (Gn), duration of Gn used, total dose of Gn and endometrial thickness on HCG Day were significantly lower in GnRH-ant group than those in GnRH-a group (*P < 0.01*), whereas the LH level on HCG Day was higher in GnRH-ant group (*P < 0.01*). Then, we investigated the clinical outcomes from these two groups (Table 3). There were no differences in the number of oocytes retrieved, 2PN fertilization rate, 2PN cleavage rate, high quality embryo rate and high quality blastulation rate between these two groups (*P > 0.05*). However, GnRH-ant group showed remarkably decreased rate of HCG positive, embryonical implantation and clinical pregnancy when compared to GnRH-a group (*P < 0.01*), implying the worse clinical outcomes in GnRH-ant group.

**Table 2:** Comparison of the effect of ovulation promotion between two groups of patients

	GnRH-ant (*n* = 563)	GnRH-a (*n* = 2252)	*P*-value
Initial dose of Gn (IU)	212.92 ± 75.17	177.83 ± 69.62	0.0000
Duration of Gn used (d)	9.11 ± 1.47	11.19 ± 1.92	0.0000
Total dose of Gn (IU)	2132.1 ± 631.08	2391.2 ± 857.17	0.0000
Endometrial thickness on HCG day (mm)	7.51 ± 2.27	9.09 ± 2.61	0.0000
E_2_ on HCG day (pg/mL)	2041.17 ± 1170.34	2080.32 ± 1022.21	0.4728
LH on HCG day (mIU/mL)	2.14 ± 1.51	1.06 ± 0.94	0.0000
P on HCG day (ng/mL)	0.86 ± 1.21	0.8 ± 1.46	0.3471

Note: The data were expressed as mean ± SD

Abbreviations: *Gn* gonadotropin, *HCG* Human Chorionic Gonadotropin, *LH* luteinizing hormone, *E*_2_ estradiol, *P* progesterone

**Table 3:** Comparison of clinical outcomes between two groups of patients

	GnRH-ant (*n* = 563)	GnRH-a (*n* = 2252)	*P*- value
Number of oocytes retrieved	11.26 ± 3.65	11.44 ± 3.58	0.8006
2PN fertilization rate (%)	60.3	61.64	0.2813
2PN cleavage rate (%)	95.75	95.82	0.8686
High quality embryo at day 3 rate (%)	28.59	28.22	0.6765
High quality blastulation rate (%)	36.2	34.01	0.1091
Number of transfer embryos	1.91 ± 1.7	1.88 ± 1.83	0.1627
HCG positive rate (%)	62.95	71.72	0.0018
Implantation rate (%)	40	47.62	0.0007
Clinical pregnancy rate (%)	55.41	64.04	0.0038
Live birth rate (%)	49.64	50.64	0.2153

Abbreviations: *PN* pronucleus, *HCG* Human Chorionic Gonadotropin

### GnRH-ant reduced the proliferation and induced the apoptosis of human isolated ESCs

**Fig. 1:**
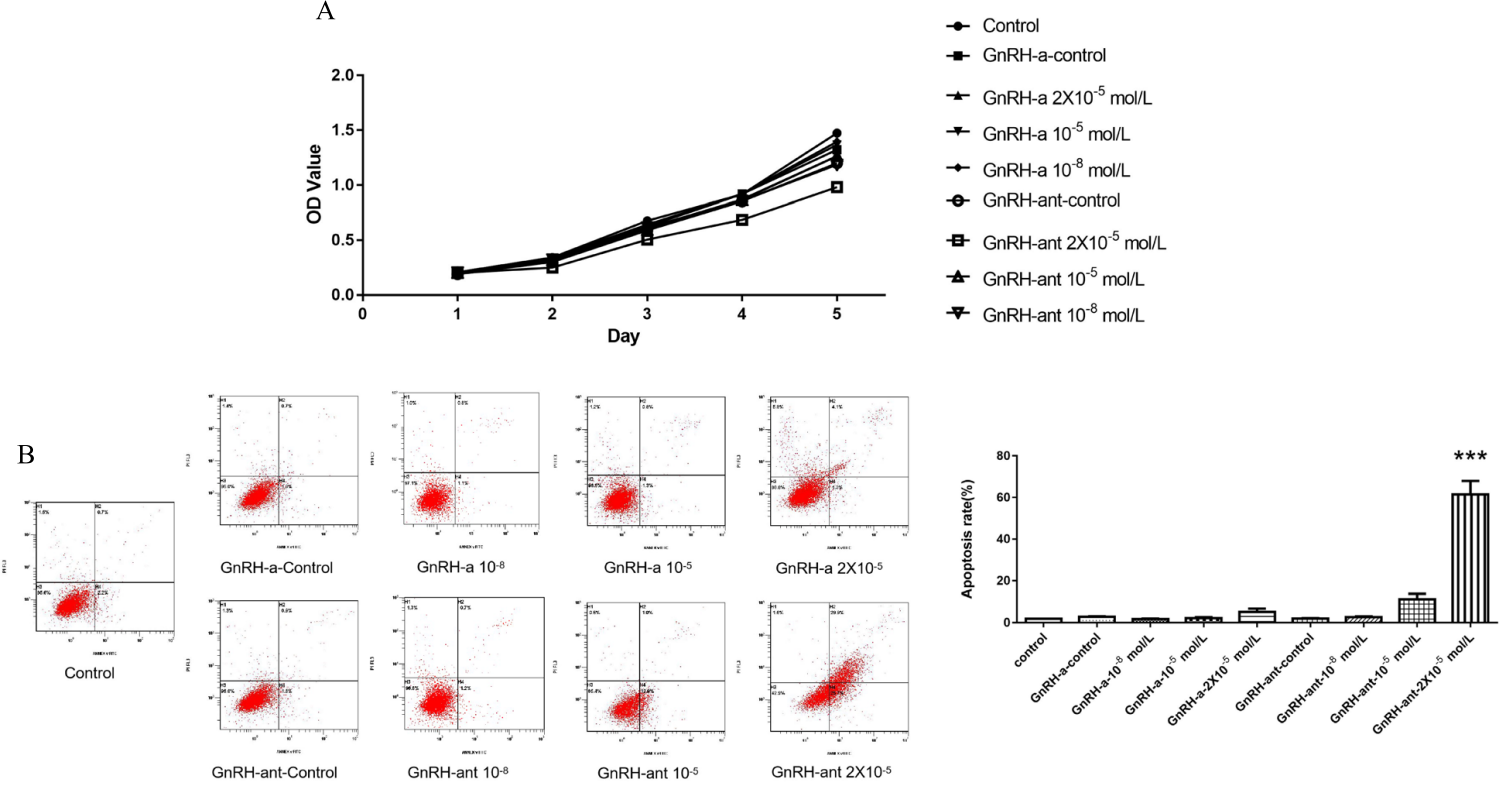
Comparison of the growth ability of human isolated ESCs after treating with different concentrations of GnRH-ant and GnRH-a (10^− 8^, 10^− 5^ and 2 × 10^− 5^ mol/L). **A** The proliferation of ESCs was investigated by using CCK8 assay. No difference was found in the proliferation of ESCs at concentration of 10^− 8^ and 10^− 5^ mol/L between GnRH-ant and GnRH-a group. However, when the concentration was added up to 2 × 10^− 5^ mol/L, the proliferation capacity of ESCs in GnRH-ant group was significantly restrained compared to those in GnRH-a group. **B** Flow cytometry was used to analyze the apoptosis of ESCs. Similarly, the apoptotic level of ESCs was remarkably increased when the concentration at 2 × 10^− 5^ mol/L. Each group of experiment was repeated for three times. (Control group: ESCs was treated only with culture medium. GnRH-ant-control group: ESCs was treated with GnRH-ant solvent. GnRH-a-control group: ESCs was treated with GnRH-a solvent.) (*** *p < 0.001*)

CCK8 and Flow cytometry were used to investigate the effects of GnRH-ant on the growth ability of human isolated ESCs, which was confirmed by immunohistochemical analysis (Supplementary Fig. 1). Little differences were found in the concentrations of GnRH-ant and GnRH-a at 10^− 8^ and 10^− 5^ mol/L. When the concentration was elevated to 2 × 10^− 5^ mol/L, GnRH-ant obviously reduced the proliferation and induced the apoptosis of ESCs (Fig. 1).

### GnRH-ant decreased the expression of c-kit receptor in human isolated ESCs

**Fig. 2:**
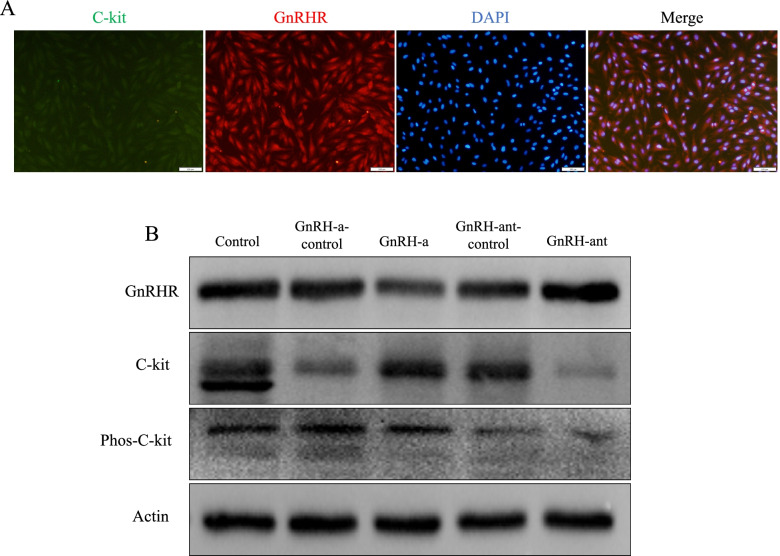
The expression of GnRH receptor (GnRHR) and c-kit receptor was detected by Immunofluorescence (**A**) and Western Blot (**B**), respectively. Furthermore, exposure of ESCs to the GnRH-ant at 2 × 10^− 5^ mol/L significantly decreased the expression of c-kit receptor followed by the attenuated phosphorylation of c-kit receptor (**B**). Each group of experiments was repeated three times. (Control group: ESCs were treated only with culture medium. GnRH-ant-control group: ESCs were treated with GnRH-ant solvent. GnRH-a-control group: ESCs were treated with GnRH-a solvent.) (*n* = 4 per lane)

As shown in the results of immunofluorescence staining, GnRH receptor and c-kit receptor were co-expressed on human isolated ESCs, indicating a possible potential regulatory relationship between these two receptors (Fig. 2A). After treating human isolated ESCs with the concentration of GnRH-ant and GnRH-a at 2 × 10^− 5^ mol/L, we found significantly reduced expression of c-kit receptor and phosphorylation c-kit receptor in GnRH-ant group (Fig. 2B), suggesting c-kit signaling was attenuated by GnRH-ant.

### C-kit receptor regulated the growth ability of human isolated ESCs through AKT signaling pathway

**Fig. 3:**
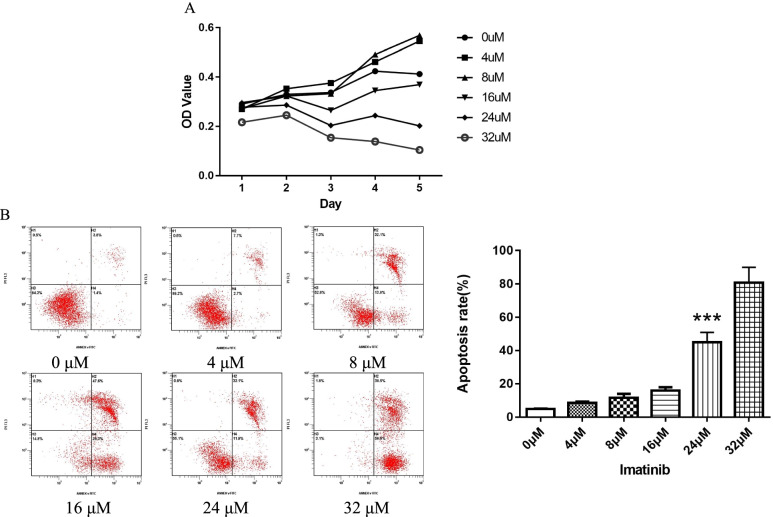
Investigation of the proliferation (**A**) and apoptosis (**B**) of human isolated ESCs after treating with different concentrations of Imatinib (0 μM, 4 μM, 8 μM, 16 μM, 24 μM, 32 μM). With the increase of drug concentration, the proliferation of ESCs was distinctly repressed (**A**) and the apoptotic level of ESCs was dramatically elevated (**B**). Each group of experiment was repeated for three times (*** *p < 0.001*)

**Fig. 4:**
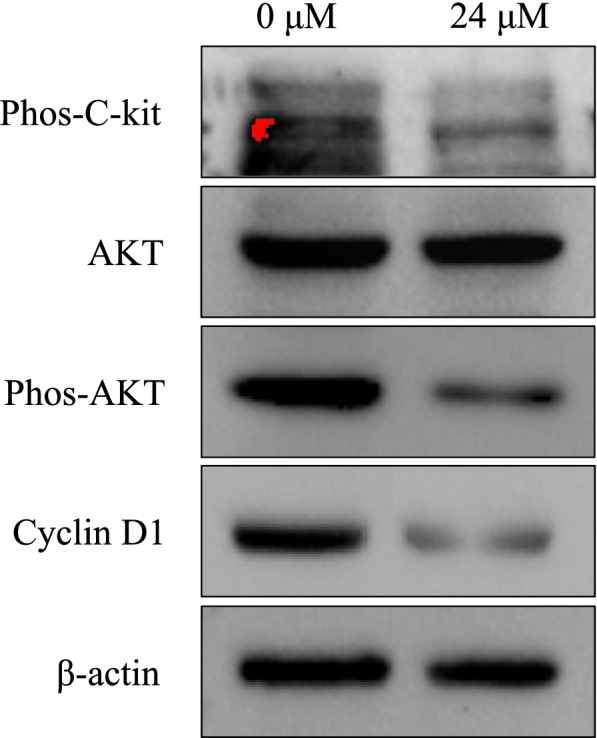
After inhibiting the activation of c-kit receptor by treating ESCs with Imatinib (24 μM), the phosphorylation of AKT and the expression of Cyclin D1 were significantly diminished. Each group of experiment was repeated for three times (*n* = 4 for per lane)

Cultured human isolated ESCs were treated with different concentrations of imatinib (0 μM, 4 μM, 8 μM, 16 μM, 24 μM, 32 μM) to confirm the ideal dosage required to suppress the c-kit receptor activation. The results showed that with the increase concentration of imatinib, the reduction of proliferation and induction of apoptosis in ESCs were increasingly notable (Fig. 3). Due to most of ESCs were dead at 32 μM, we selected 24 μM concentration as the ideal dosage to treat ESCs. The results showed that after inhibiting the c-kit receptor activation by 24 μM imatinib in ESCs, the phosphorylation of AKT and the expression of cyclin D1 were significantly decreased (Fig. 4).

## Discussion

In the normal menstrual cycle, endometrium would go through three phases: menstrual period, proliferative period and decidual period. During the decidual phase, the endometrium transformed into a receptive tissue that was suitable for embryo implantation [23, 24]. In this process, ESCs were the main cell type providing nutrition for the implanting embryos [25]. Therefore, the abundant numbers of ESCs would directly decide the success of embryo implantation. In the present study, although there were no differences in embryo quality between GnRH-ant and GnRH-a group, GnRH-ant group produced lower rates of HCG positive, embryo implantation and clinical pregnancy. It was worth noting that a thinner endometrial thickness was observed in GnRH-ant group, suggesting that the appropriate endometrial thickness might be conducive to embryo implantation. Similar with our findings, many recent studies also found that the thinner endometrium was evidently related to the worse clinical outcomes in fresh and frozen-thaw embryo transfer [26–29]. One of the reasons resulting in worse clinical outcomes in GnRH-ant group might be the adverse effect of GnRH-ant on growth ability of human ESCs. Accordingly, we isolated human ESCs from human endometrial tissues and treated cells with different concentrations of GnRH-analog to investigate the effects of GnRH-ant on the growth ability of ESCs. GnRH-ant significantly depressed the proliferation and promoted the apoptosis of ESCs, confirming the adverse influences of GnRH-ant on ESCs growth. However, how GnRH-ant negatively regulates the growth of ESCs is still unclear.

Previous studies have reported the positive effects of c-kit receptor on growth ability of multiple kinds of cells [30–33]. Furthermore, recent studies have found the expression of c-kit receptor on animal embryo and endometrium, and proposed its important role in the process of embryo implantation [15]. Surprisingly, we found the expression of c-kit receptor on human ESCs, indicating that c-kit receptor might also regulate the growth ability of human ESCs. Whereafter, we treated human ESCs with imatinib (c-kit receptor inhibitor) and found that after inhibiting the activation of c-kit receptor by imatinib, ESCs showed remarkably decreased proliferation capacity and increased apoptosis level. Moreover, the phosphorylation of AKT and the expression of cyclin D1, which were closely associated with cell growth, were significantly weakened. These findings supported that activating c-kit receptor could accelerate the growth ability of human ESCs.

Based on the above findings, it is reasonable to speculate that whether the adverse effects of GnRH-ant on human ESCs growth ability is correlated with the inactivation of c-kit receptor? To further verify our hypothesis, we subsequently treated ESCs with GnRH-ant at 2 × 10^− 5^ mol/L for 120 h. The results showed that GnRH-ant significantly suppressed the expression of c-kit receptor followed by the attenuated phosphorylation of c-kit receptor, implying that GnRH-ant could weaken the growth ability of human ESCs by impairing the activation level of c-kit receptor through suppressing c-kit receptor expression. Notably, recent study directed by Chen et al. have demonstrated that GnRH-ant could disrupt human endometrial epithelial cells (EECs) migration by reducing the CKB expression, which altered endometrial receptivity [34]. Complementally, our findings showed another negative effect of GnRH-ant on ESCs, which was the repression of ESCs growth capacity. Even so, the whole effects of GnRH-ant protocol on endometrium of women undergoing IVF-ET were still unclarified. Hence, further researches related the molecular mechanism are needed.

Normally, the levels of endogenous GnRH in peripheral blood circulation were very low, thus, the biological functions resulting from the combination of GnRH and GnRH receptor outside the pituitary were very fewer [35]. Furthermore, GnRH-ant has greater affinity to GnRH receptor than GnRH and GnRH-a [36]. Therefore, GnRH-ant was more likely to combine with GnRH receptor outside the pituitary, such as endometrium [37]. Actually, several studies have found the time- and dose dependent growth suppressive functions of GnRH-ant in various cancer cell lines, including endometrium cancer, ovary cancer and breast cancer [38, 39]. Although the specific molecular mechanisms were still unclear, it was reasonable to believe that the signaling mechanisms of GnRH- receptor in pituitary were not involved in inhibitory growth effects of GnRH-ant in cancer cells. Although the present study demonstrated the inhibitory effects of GnRH-ant on ESCs growth ability, these mechanisms were not enough to fully explain the results of women undergoing the GnRH-ant protocol. Therefore, more in-depth studies were still needed.

## Conclusion

In brief, GnRH-ant attenuated the activation level of c-kit receptor by decreasing its expression in ESCs. Subsequently, this impaired effect could further inhibit the phosphorylation of AKT and lessened the expression of cyclin D1, which resulted in the reduction of growth ability of ESCs. The present study provides a new insight into the role of GnRH-ant and suggests that the adverse effects of GnRH-ant protocol on endometrium should be considered before using in IVF-ET.

## Supplementary Information

**Additional file 1: Supplementary Figure 1.** ESCs was verified by immunohistochemical analysis. As shown in results, the expression of vimentin but not cytokeratin was found in human isolated ESCs.

## Data Availability

All data generated and analyzed in this study are included in this published manuscript.

## Abbreviations

GnRH-ant: Gonadotropin-releasing hormone antagonist
GnRH-a: Gonadotropin-releasing hormone agonist
GnRHR: Gonadotropin-releasing hormone receptor
ESCs: Endometrial stromal cells
EECs: Endometrial epithelial cells
IVF-ET: In vitro fertilization-embryo transfer
RTK: Receptor tyrosine kinase
BMI: Body mass index
AFC: Antral follicle count
AMH: Anti-mullerian hormone
rhFSH: Recombinant human FSH
