# Supplementary figures and images for: GnRH antagonist weakens endometrial stromal cells growth ability by decreasing c-kit receptor expression
Source: Reprod Biol Endocrinol. 2022 Feb 4;20:29. doi: 10.1186/s12958-021-00886-y (PMC8815158; doi:10.1186/s12958-021-00886-y)

**Control**

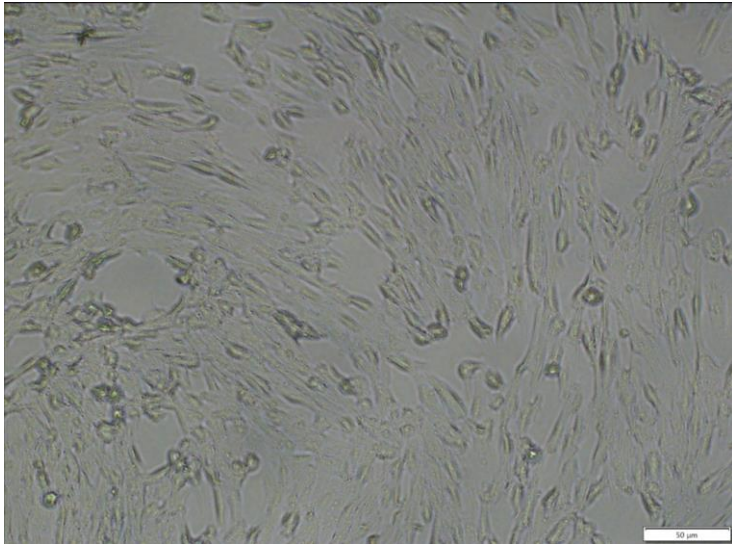

**Vimentin**

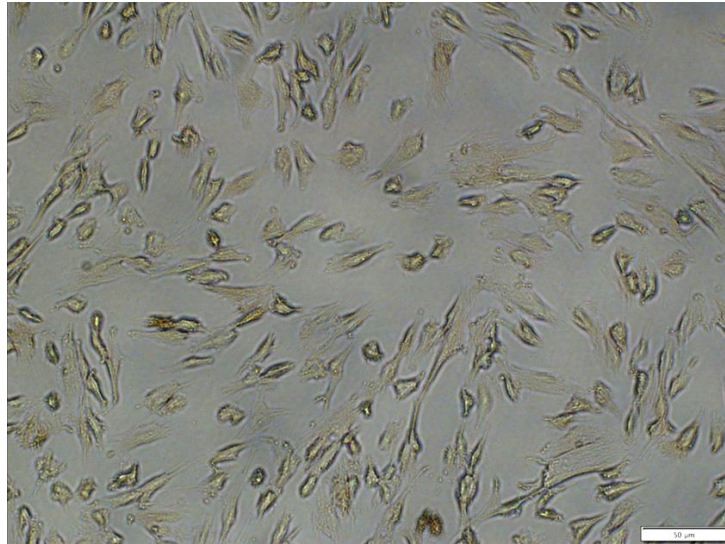

**Cytokeratin**

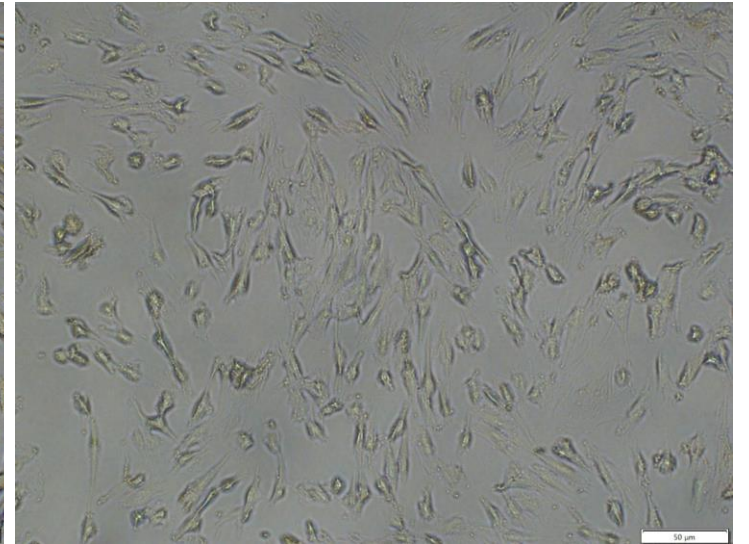

Supplement: Supplementary file 1 — Additional file 1: Supplementary Figure 1. ESCs was verified by immunohistochemical analysis. As shown in results, the expression of vimentin but not cytokeratin was found in human isolated ESCs. [file 12958_2021_886_MOESM1_ESM.pdf]
